# Material practices for meaningful engagement: An analysis of participatory learning and action research techniques for data generation and analysis in a health research partnership

**DOI:** 10.1111/hex.12598

**Published:** 2017-08-25

**Authors:** Mary O'Reilly‐de Brún, Tomas de Brún, Catherine A. O'Donnell, Maria Papadakaki, Aristoula Saridaki, Christos Lionis, Nicola Burns, Chris Dowrick, Katja Gravenhorst, Wolfgang Spiegel, Chris Van Weel, Evelyn Van Weel‐Baumgarten, Maria Van den Muijsenbergh, Anne MacFarlane

**Affiliations:** ^1^ Discipline of General Practice National University of Ireland Galway Ireland; ^2^ Centre for Participatory Strategies Galway Ireland; ^3^ General Practice and Primary Care University of Glasgow Glasgow UK; ^4^ Clinic of Social and Family Medicine Faculty of Medicine University of Crete Heraklion Greece; ^5^ University of Liverpool Liverpool UK; ^6^ Centre for Public Health Medical University of Vienna Wien Austria; ^7^ Department of Primary and Community Care Radboud University Nijmegen Medical Centre Nijmegen Gelderland Netherlands; ^8^ Graduate Entry Medical School and Health Research Institute University of Limerick Limerick Ireland

**Keywords:** health research partnerships, migrant health, participatory research, public and patient involvement

## Abstract

**Background:**

The material practices which researchers use in research partnerships may enable or constrain the nature of engagement with stakeholder groups. Participatory learning and action (PLA) research approaches show promise, but there has been no detailed analysis of stakeholders’ and researchers’ experiences of PLA techniques for data generation and co‐analysis.

**Objectives:**

To explore stakeholders’ and researchers’ experiences of PLA techniques for data generation and co‐analysis.

**Design:**

The EU RESTORE implementation science project employed a participatory approach to investigate and support the implementation of guidelines and training initiatives (GTIs) to enhance communication in cross‐cultural primary care consultations. We developed a purposeful sample of 78 stakeholders (migrants, general practice staff, community interpreters, service providers, service planners) from primary care settings in Austria, England, Greece, Ireland and The Netherlands. We used speed evaluations and participatory evaluations to explore their experiences of two PLA techniques—Commentary Charts and Direct Ranking—which were intended to generate data for co‐analysis by stakeholders about the GTIs under analysis. We evaluated 16 RESTORE researchers’ experiences using interviews. We conducted thematic and content analysis of all evaluation data.

**Results:**

PLA Commentary Charts and Direct Ranking techniques, with their visual, verbal and tangible nature and inherent analytical capabilities, were found to be powerful tools for involving stakeholders in a collaborative analysis of GTIs. Stakeholders had few negative experiences and numerous multifaceted positive experiences of meaningful engagement, which resonated with researchers’ accounts.

**Conclusion:**

PLA techniques and approaches are valuable as material practices in health research partnerships.

## 1 INTRODUCTION

Involving patients and communities in health research partnerships is consistent with international policies and is recommended for ethical and instrumental reasons.^1^, ^2^ It is increasingly a requirement for research funding in many countries. There is a long‐standing awareness that meaningful involvement is a genuine challenge.^3^, ^4^ Recent reviews show persisting concerns that current practice is tokenistic.^5^

Our working definition of “meaningful engagement” is an experience of partnership in research that is collegial, inclusive and active for participants. Meaningful engagement reduces asymmetries of power, encourages participants’ ownership of the project and enables participants’ authentic perspectives to emerge clearly in research outcomes.^6^, ^7^, ^8^

“Material practices,” such as the types of methods and techniques that researchers use to involve stakeholders, can enable or constrain participation in research.^9^ Therefore, while there are valid concerns about a “flight to empiricism” and an overemphasis on “how to” manuals,^4^, ^10^ it is important to determine what methods and techniques are used to frame the interactional and relational nature of partnerships. This will allow identification of material practices which minimize tokenism and enhance opportunities for meaningful engagement.

Research to identify the best methods to achieve meaningful engagement is currently lacking.^11^ Boote et al.^12^ reported that group meetings were the most common method used to engage the public. Workshops, meetings and focus groups were identified as common methods of engagement in three other reviews.^13^, ^14^, ^15^ Domecq et al.^11^ found that the most common methods in use were focus groups, interviews and surveys.

Tierney et al.'s^5^ review of service user involvement in academic primary care also found that interviews and focus groups were commonly employed. It reported examples of studies which had used methods from the field of participatory health research and found that the use of participatory methods was more congruent with stated aspirations for meaningful engagement than “standard” research methods were.

Participatory health research is an overarching term that refers to “bottom‐up” research approaches specifically designed for stakeholder involvement in research partnerships. These include, among others, participatory research (PR),^16^, ^17^, ^18^ participatory action research (PAR),^19^, ^20^ community‐based participatory research,^21^, ^22^ participatory rural appraisal (PRA)^8^, ^23^, ^24^ and participatory learning and action (PLA).^25^, ^26^ All share a democratic ethos, are strongly committed to meaningful engagement by stakeholders and promote research partnerships that strengthen relations between academy and community. Participatory approaches emphasize the need for stakeholders’ active engagement across the full range of research activities, including data generation and data analysis.

Participatory approaches face challenges, such as the need to see community participation as a long‐term process of implementation and support for improved health outcomes^27^ and the fact that many professional health researchers may be unprepared for the reversals of power and hierarchical relationships that a participatory approach may require.^28^ Notwithstanding these challenges, there is consistent evidence that participatory approaches provide added value in terms of shaping the purpose and scope of research, improving research implementation and enhancing both the interpretation and the application of the research outcomes.^29^ Furthermore, participatory approaches offer a range of interesting and interactive material practices and techniques. PLA is noteworthy in this regard. This is a form of action research rooted in the interpretive and emancipatory paradigms.^25^, ^26^ Based on the work of Robert Chambers, PLA is a methodology which offers a practical approach to research where asymmetries of power may exist.^7^, ^8^, ^23^, ^24^, ^25^, ^30^, ^31^ It involves a combination of a *PLA mode of engagement* and *PLA techniques*. A *PLA mode of engagement* aims to create a trusting relational environment, a “safe space” where stakeholders are encouraged to respect a diversity of views and experiences, and to learn from each other's perspectives.^30^, ^31^ All stakeholders are considered to possess expert knowledge about their own lives and conditions which they bring to the “stakeholder table” for a PLA brokered dialogue, where, using various PLA techniques, implicit knowledge becomes explicit and much that otherwise might remain hidden emerges.

*PLA techniques* evolved originally from PRA and are based on a shared stock of ideas and experiences from participatory trainers and stakeholders around the globe. They continue to be adapted to specific contexts as required.^31^ The techniques are recognizable as PLA techniques because they are explicitly designed to be active, inclusive, user‐friendly and democratic. They are visual and tangible, meaning that they are used to generate physical maps, charts and diagrams (described further under Methods). Generation and co‐analysis of data go hand in hand and are best understood as a *structured, integrated process*. Stakeholders’ priorities and perspectives are meant to guide the generation and co‐analysis of data about the issue being explored, with researchers acting as catalysts rather than directors or top‐down decision‐makers.

There are some recent positive examples of PLA applied to primary care health research. These studies describe meaningful involvement of migrants and other stakeholders in the development of a guideline to improve communication in cross‐cultural consultations;^32^, ^33^ involvement of people with aphasia, speech and language therapy educators and students in the evaluation of community services for people with aphasia;^34^, ^35^ and involvement of a variety of marginalized groups (sex workers, homeless people, Irish Travellers, migrants and drug users) in the identification of priorities for primary care team activities.^36^ However, there has been no detailed analysis of stakeholders’ or researchers’ experiences of PLA techniques for data generation and co‐analysis used within a PLA‐brokered dialogue. Such an analysis would provide important empirical data about the ways in which PLA techniques are experienced as material practices^3^, ^9^ and how they shape interactional and relational aspects of health research partnerships.

In this paper, we describe the use of two PLA techniques (Commentary Charts and Direct Ranking) used for data generation and co‐analysis, and the perceived utility of these by various stakeholders and researchers involved in a recent European primary health‐care implementation project.

## 2 METHODS

### 2.1 Study setting: the RESTORE project

RESTORE (Research into Implementation Strategies to Support Patients of Different Origins and Language Background in a Variety of European Primary Care Settings) was an EU‐funded primary health‐care research project that ran from 2011 to 2015. The objective of RESTORE was to investigate and support the implementation of guidelines and training initiatives (GTIs) intended to enhance communication in cross‐cultural primary care consultations. This qualitative, comparative case study^37^ involved diverse stakeholders across five primary care settings: Austria, England, Greece, Ireland and The Netherlands (see File S1 for a description of the five settings). A sixth research team in Scotland focused on policy‐related implications of the study. The choice of these six countries matched the academic teams who developed the proposal and intentionally included countries with diverse primary health‐care systems. Ethical approval was granted by respective national committees.

A detailed description of the study protocol is available elsewhere.^38^ For the purpose of this study, we emphasize that RESTORE comprised three stages of fieldwork.

1. *Stage 1*. Stakeholders were informed about RESTORE and invited to participate. Researchers mapped GTIs that were available in each RESTORE project country.^39^
2. *Stage 2*. Stakeholders examined a set of these GTIs that showed potential for implementation in their country and selected one that they deemed most suitable or relevant for their local primary care setting.^40^
3. *Stage 3*. Stakeholders successfully adapted their selected GTI at a local level and worked on its implementation, with evidence of some impact on daily practice.^41^

RESTORE was the overall setting in which we explored and evaluated stakeholders’ and researchers’ experiences of two PLA techniques. These techniques were employed during Stage 2 and were intended to enable stakeholders (migrants, general practice staff, community interpreters, service providers, service planners and others) to work collaboratively and with RESTORE researchers to select a GTI for their local primary care setting.

First, we describe sampling and recruitment for RESTORE—this is the sample for the evaluation reported in this paper. We then describe the two specific PLA techniques employed in RESTORE and the methods used to evaluate stakeholders’ and researchers’ experiences of these techniques. Finally, we present our analysis of the evaluation data.

### 2.2 Sampling and recruitment

In Stage 2, we used a combination of purposeful and network sampling to identify and recruit 78 stakeholder representatives across five research sites. A geographically defined area (district) was selected in each partner country. Selection was pragmatic, based on researchers’ knowledge of groups working in the district and proximity to the research teams, to facilitate data collection. Eligible organizations/agencies were those involved in primary health‐care planning and delivery (eg health‐care centres, regional health authorities) as well as those addressing migrant health issues (eg non‐governmental organizations focused on migrants). The aim was to identify individuals who were decision‐makers (eg health authority service planners and policymakers), service providers (eg general practitioners (GPs), primary care staff, community interpreters) or service users (ie migrants using local primary care services).

In line with standard ethical procedures, stakeholders in all countries were provided with information leaflets and signed consent forms prior to participating in fieldwork sessions.

### 2.3 PLA data generation and co‐analysis in RESTORE

Two members of the RESTORE consortium in the Irish team (practitioner/trainers with over 25 years’ international experience in PLA research and training in diverse cultural and social settings)^26^, ^32^, ^33^, ^42^, ^43^, ^44^ led the design of PLA in RESTORE. They provided training and standardized fieldwork protocols which enabled researchers to facilitate PLA in a consistent and rigorous manner across research sites.

The PLA process for RESTORE was based on a *PLA mode of engagement* and *PLA techniques* in a PLA‐brokered dialogue between stakeholders. One of the striking features of PLA in general is the highly *visual* nature of the techniques used. Stakeholders work together to generate maps, charts and diagrams which function as powerful reference points (data displays) as they engage in *verbal* interaction, discussing, questioning, and learning from each other's perspectives, adding new data to maps and charts.^8^, ^32^, ^44^, ^45^, ^46^, ^47^ The *inherent analytical capabilities* of PLA techniques aim to enable stakeholders to assess, correlate, categorize and/or prioritize data they are co‐generating. PLA techniques, therefore, have the capacity to facilitate meaningful engagement that *automatically incorporates* co‐generation and co‐analysis of data “by” and “with” stakeholders. In a very practical way, then, stakeholders using PLA techniques engage in a structured, integrated, *visual*‐*verbal‐tangible* process of co‐generating and co‐analysing data which produces *visual‐tangible* results.^6^, ^25^, ^32^, ^43^ This activity can appeal to a wide range of stakeholder groups, including those where literacy and/or numeracy challenges may feature, as a key aim is to ensure that stakeholders/stakeholder groups do not become disenfranchised during the research process. Data co‐generation and co‐analysis may occur during a single PLA session, or iteratively and in successive waves of fieldwork and/or data‐generation encounters during PLA research, bringing stakeholders’ unique knowledge and insights to bear on emerging findings. This ensures that stakeholders’ perspectives influence the conduct and trajectory of research and research outcomes—a key hallmark of meaningful engagement.^3^, ^25^

The two PLA techniques we used in RESTORE in Stage 2 were Commentary Charts and Direct Ranking. A Commentary Chart is an interactive knowledge‐exchange, knowledge‐enhancement technique which allows stakeholders to exchange differential knowledge, expertise and perspectives. Box 1 provides information on generic steps for facilitating Commentary Charts, based on the expertise of the RESTORE project PLA trainers.

Box 1 **Step‐by step guidance for the use of PLA Commentary Charts and Direct Ranking Techniques**

**Commentary Chart**

- Stakeholders record key data on Post‐It notes about the issue of interest. This provides a visual representation of their co‐generated data.
- The Post‐Its are assigned to the relevant category on the chart. These categories may be determined before or during the sessions. This is the start of the co*‐*analysis process.
- Researchers and stakeholders consider and discuss the emerging and completed Commentary Chart. This process automatically incorporates co‐generation and co‐analysis of data “by” and “with” stakeholders.
- Researchers and stakeholders continue the co‐analysis with a visual‐verbal‐tangible process of “interviewing” the chart. The emphasis is on looking at the Commentary Chart and encouraging stakeholders to share their unique knowledge and insights, to exchange differential knowledge by asking: *Does the Commentary Chart make sense? Are stakeholders comfortable with their data display? Is there anything striking/odd about the data display? Are diverse views sufficiently and accurately represented? Does anything need to be added as we reflect on the Chart? Are stakeholders willing and content to “sign off” on the Chart? Can it now be presented to another stakeholder group (as needs be) for discussion and development?*

**Direct Ranking**

- Physical objects and/or images are selected to represent the issues/entities being ranked. This provides a visual focus for the co‐analysis process.
- Stakeholders place the selected images randomly on a large flipchart sheet, to give each image equal visual “weight” and importance.
- Stakeholders engage in co‐analysis and clarify what the ranking criterion will be.
- Stakeholders discuss each object/image in relation to the agreed ranking criterion, listening, learning, questioning, reflecting and assessing, thus continuing the integrated processes of data generation and analysis.
- When discussion is complete, each stakeholder is provided with an equal number of “votes” (eg paper clips, coins, matches).
- Stakeholders distribute their votes across the images.
- Stakeholders count the number of votes assigned to each image.
- Results are double‐checked, recorded in numerical form on “Post‐It” notes and attached to the relevant images.
- Stakeholders draw a line down the centre of the flipchart sheet and place the images on the line: highest scoring image at the top, lowest at the bottom, others in between in positions dictated by number of votes accrued.
- Researchers and stakeholders continue the co‐analysis with a visual‐verbal‐tangible process of “interviewing” the results of the Direct Ranking process. The emphasis is on looking at the Direct Ranking chart and encouraging stakeholders to share their unique knowledge and insights, to exchange differential knowledge by asking: does it make sense? *Do stakeholders feel comfortable with the outcome? Is there anything striking/odd about the result? Having been decided by democratic vote, is the result definitely acceptable? Are stakeholders willing and content to “sign up” to the result?*

**Figure 1:**
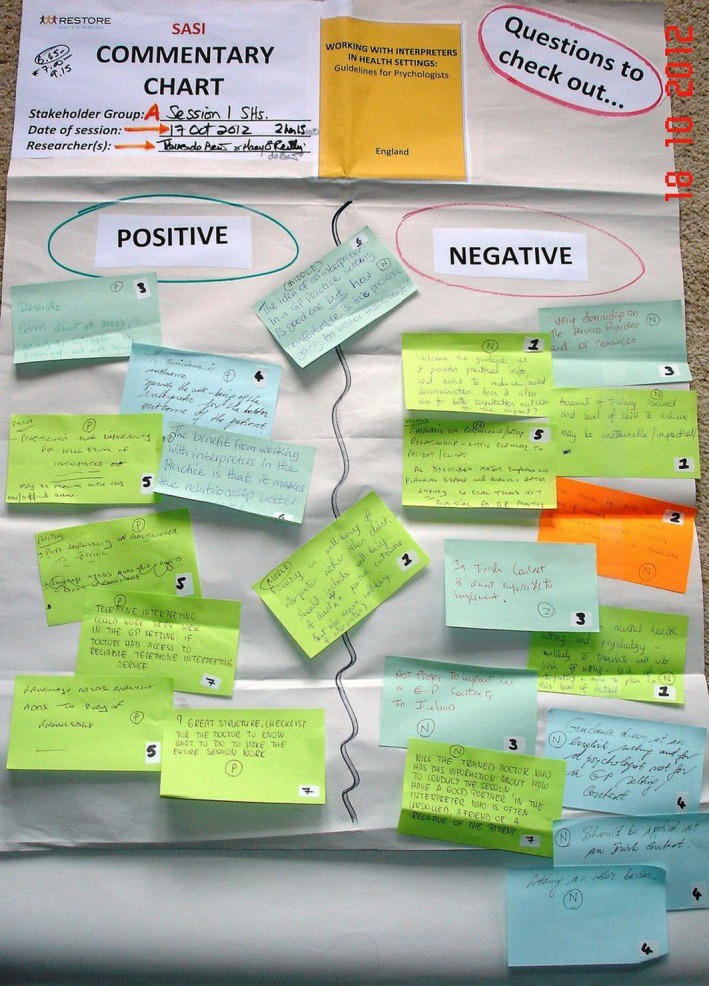
Commentary Chart—Ireland

In RESTORE, PLA Commentary Charts were used to facilitate dialogue among stakeholders about the GTIs mapped in Stage 1. Full‐text copies, summaries and/or PowerPoint presentations of the identified GTIs were made available to stakeholders. The Commentary Charts comprised three analytical categories—”positive aspects of the GTI,” “negative aspects of the GTI” and “questions to be checked out.” Stakeholders recorded their key deliberations, perspectives and insights about each GTI on the Commentary Chart (see Figure 1). For example, in Ireland, stakeholders met in one large group for each of five PLA sessions. They had identified a “local set” of five GTIs and co‐generated five separate Commentary Charts. Where several stakeholder groups met separately (eg Austria) or were geographically dispersed (eg The Netherlands), charts were computerized and circulated around stakeholder groups by email, iteratively accruing additional data. On occasion, researchers took physical charts from one stakeholder group to the next, and data were added incrementally. As Commentary Charts “travelled” around stakeholder groups, the knowledge‐exchange and knowledge‐enhancing process continued.

The intended practical outcome expected of Commentary Charts in RESTORE was that they would present a visual, tangible data display of stakeholders’ knowledge, expertise and perceptions about the sets of GTIs. Stakeholders could then review the data display and continue their co‐analysis activity as they began to use Direct Ranking to select a single GTI for implementation at local level.

**Figure 2:**
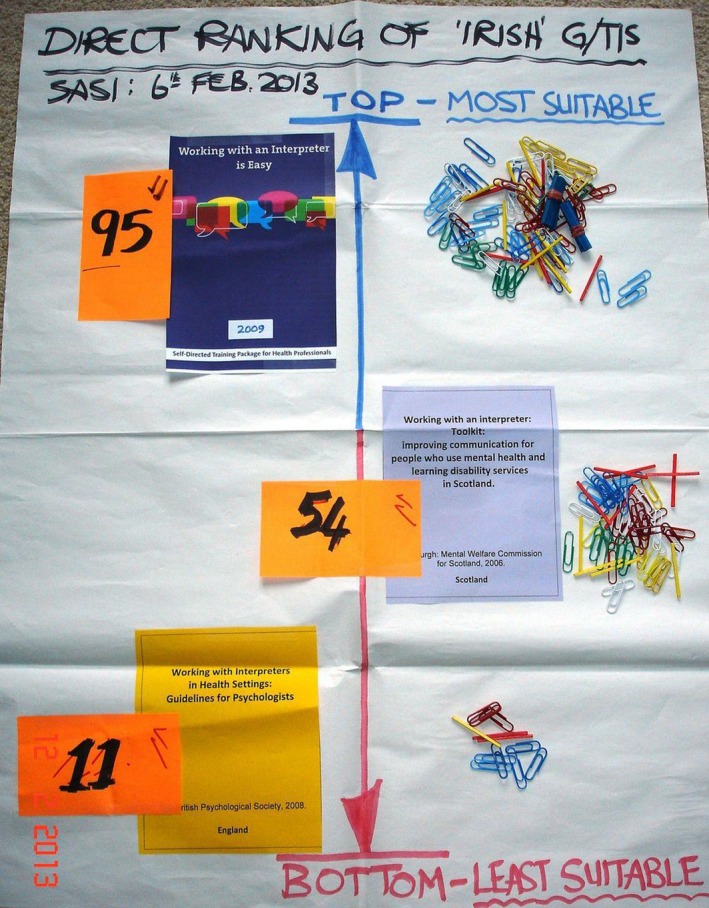
Direct Ranking result—Ireland

Direct Ranking is an interactive technique for identifying priorities or preferences in a democratic manner. It yields a visual result in chart form. Box 1 provides a summary of the generic steps for this technique. In RESTORE, the specific application of Direct Ranking was to produce a clear, documented democratic result—a single GTI for implementation that stakeholders are willing to “sign up to” for Stage 3. The images selected to represent each GTI were photographs of the front covers of GTIs. The agreed ranking criterion was “Prioritize the GTIs in terms of the most‐to‐least suitable for implementation in our general practice setting and context.” Stakeholders had equal voting power as they had 20 paper clips each (see Figure 2). The intended practical outcome expected of Direct Ranking Charts in RESTORE was that they would present a visual, tangible data display of stakeholders’ decision about which GTI was considered most suitable for implementation in their setting.

Across research sites, the majority of PLA sessions involving Commentary Charts and Direct Ranking were each 2‐3 hours in duration. The completed paper‐based charts were computerized to preserve them and to make them readily available for further analysis.

### 2.4 Evaluation of the use of PLA techniques in RESTORE

Stakeholders at all sites participated in qualitative “speed” evaluations (SEs) to document their experiences of involvement in their PLA sessions. A “speed evaluation” is a brief verbal (digitally recorded) or written evaluation, which provides an opportunity for stakeholders to describe experiences in their own words and suggest areas for improvement. It allows researchers to “take the temperature” of the group, to build on positives, and, where possible, to plan suggested improvements for forthcoming PLA sessions. Speed evaluations usually occur at the close of a PLA process or session. Participants respond to an open‐ended question in a rapid, interactive and spontaneous way.

For example, on completion of Commentary Charts, the question was as follows: “During this session, we worked together in a participatory way: listening, learning, creating our Commentary Charts. In a word or phrase, please comment on *your* experience of this.”

Stakeholders at the Irish site also completed a participatory evaluation (PE) because the facilitators there had extensive experience of this technique. This form of more in‐depth collaborative evaluation is judgement‐based and may be formative or summative. Stakeholders responded to questions focused on Direct Ranking and their experiences of engagement in the PLA process. An “open category” question also invited stakeholders to comment on any aspect of their experience, especially if there were any improvements and/or changes that could be made.

Speed and participatory evaluations were either noted or audio‐taped and transcribed at each site, and collated as reports by the researchers. We had ethical approval to include data from all of these reports in the analysis for this paper, apart from the data from the English site.

All academic researchers involved in PLA fieldwork participated in in‐depth reflection interviews (R Int), conducted by the PLA trainers, at the close of Stage 2 fieldwork (n=16). Interviews were conducted face‐to‐face or by Skype; interview length ranged from 50 minutes to 1 hour 45 minutes, depending mainly on the number of researchers involved (eg Austria=1 interviewee, The Netherlands=4). The interview schedule was circulated to all teams in advance. They were asked to reflect on the use of Commentary Charts and Direct Ranking with reference to their experiences of the techniques and their observations of stakeholders’ experiences of them (see File S2 for this topic guide). Interviews were audio‐taped and professionally transcribed.

All evaluation data were collated and analysed following the principles of thematic analysis in qualitative research.^48^, ^49^ The PLA researchers at the Irish site, who have more than 25 years’ experience of qualitative interviewing, generated a “start list” of codes^50^, ^51^ derived from participatory research literature describing meaningful engagement (eg active inclusion, collaboration/collegiality, power‐sharing) and its opposite (eg exclusion/passivity, researcher‐controlled, powerlessness).^6^, ^7^, ^8^ This, augmented by repeated readings of researchers’ and stakeholders’ data, generated a final set of 33 codes. Each code was understood to incorporate its mirror or binary opposite. Data were collated under these codes to identify emerging themes.

The researchers also conducted a basic content analysis to establish the relative weighting of “positive” to “negative” evaluation comments in the final set of themes.^52^, ^53^ In keeping with our comparative case study design, the analysis of all evaluation data explored shared and differential findings across the five contexts.

Using different enquiry techniques (speed and participatory evaluations, focus group discussions and team “reflection” interviews) to explore researchers’ *and* stakeholders’ perspectives about their experiences of the same events (PLA sessions), we achieved a measure of triangulation, or cross‐validation. As per Lincoln and Guba,^49^ *prolonged involvement* of researchers in fieldwork for “*trust‐building”* and *“knowing the culture*,” coupled with *persistent observation* of stakeholders’ reactions to PLA methods and *peer debriefing* by research teams when producing regular field reports, contributed to study depth. This, in conjunction with the triangulation or cross‐validation mentioned above, contributed to and enhanced the trustworthiness, credibility and dependability of the study.^49^, ^53^

## 3 RESULTS

### 3.1 Study sample

There was appropriate representation of stakeholders at each site by gender, age group, country of origin and type of stakeholder group (see Table 1), thus validating the purposefulness of the sample.

**Table 1:** Stakeholders’ socio‐demographic characteristics

Gender	Austria	England	Greece	Ireland	Netherlands
Male	6	2	6	3	8
Female	9	7	10	8	19
Age group					
18‐30	3	2	3	0	2
31‐55	9	7	11	11	20
56 plus	3	0	2	0	5
Country of origin					
Chile	‐	‐	‐	1	‐
Congo	‐	‐	‐	1	‐
Ireland	‐	‐	‐	3	‐
Nigeria	‐	‐	‐	1	‐
Poland	‐	‐	‐	1	‐
Portugal	‐	‐	‐	1	‐
Russia	‐	‐	‐	1	‐
Netherlands	‐	‐	1	1	22
Morocco	‐	‐	‐	‐	1
Indonesia	‐	‐	‐	‐	3
Philippines	2	‐	‐	‐	1
Greece	‐	‐	13	‐	‐
Syria	‐	1	1	‐	‐
Albania	‐	‐	1	‐	‐
UK	‐	6	‐	‐	‐
Pakistan	‐	1	‐	‐	‐
Austria	7	‐	‐	‐	‐
Croatia	2	‐	‐	‐	‐
Turkey	2	‐	‐	‐	‐
Ghana	1	‐	‐	‐	‐
Benin	1	‐	‐	‐	‐
Undefined	‐	1	‐	‐	‐
Stakeholder group					
Migrant community	8	7	2	8	8
Primary care doctors	5	1	4	1	8
Primary care nurses	1	0	5	0	2
Primary care admin/management staff	1	0	1	1	6
Interpreting community	0	1	0	5	0
Health service planning and/or policy personnel	5	1	5	1	4

We monitored attendance at each session and over time. There was good, sustained involvement of stakeholders across settings for the duration of Stage 2, which involved six PLA sessions. Specifically, there were only minor variations in the sample, for example if a stakeholder could not attend due to work or personal commitments. Only two stakeholders dropped out (both from the English setting) during the fieldwork period (September 2012 to May 2013).

There was strong representation of the academic research team, as 16 of 18 individuals participated in PLA fieldwork and its evaluation (see Table 2).

**Table 2:** Description of RESTORE researchers trained in PLA

Gender	Austria	England	Greece	Ireland	Netherlands	Scotland
Male	1	1	1	1	1	0
Female	1	1	3	2	3	
Age group						
18‐30	1	1	3	0	2	
31‐55	1	0	1	3	2	1
56 plus	0	1	0	0		
Country of origin						
America			1			
Austria	2					
Denmark		1				
England		1				
Greece			3			
Ireland				3		
Scotland						1
The Netherlands					4	

### 3.2 Emergent themes

We identified five interrelated themes of stakeholders’ experiences that elucidate their positive perspective on the PLA techniques (see Table 3). Findings were relevant across countries and participant groups unless otherwise specified.

**Table 3:** Positive experiences of involvement

Analytical themes	Supporting quotes from stakeholders (SH) and researchers (R)
1. Meaningful engagement in “safe space”	*Great feeling of safety within the group. I felt comfortable to express my views and suggestions*. IRL Migrant and Community interpreter PE
	*It is not necessarily always the case to work with different professionals in this relaxed way*. AUS academic SE
	*Interaction gives the participant an opportunity to express his/her views*. NL Nurse SE
	*Service users at the other end of the [power] spectrum, if you like, who maybe are used to having little power in that type of situation, felt very protected and safe. I think it's a testimony to the process as well as everything else*. IRL Researcher #1 R Int
2a. Enhanced learning	*Brilliant process. Couldn't have predicted the variety of viewpoints and perspectives*. IRL GP SE
	*The participatory approach was very interesting as well as how we exchanged ideas and knowledge …* GR academic SE
	*The most interesting [thing] for me was that we also had the opportunity to talk to each other about our own experiences. I did not know that so many people do have the same*—*or at least familiar*—*experiences, like me*. AUS Migrant SE
	*It is definitely useful, fascinating and, to my idea, effective to analyse trainings [GTIs] together*. NL Practice Nurse SE
2b. Enhanced learning generated new understandings	*I suddenly recognised that those essential contents do not only affect migrants, or people with a migratory background, but also [other] patients*. AUS GP SE
	*Evaluating trainings with other disciplines is nice and inspiring (leads to out of the box thinking)*. NL GP SE
	*Usually, in these sessions, I think of the interpreting point of view and then of the view of the migrant service‐user but then I said, “No, I also have to [think] from the point of view of someone that helps migrants a lot [referring to another stakeholder”]*—*it's* three *hats I have to put on all of the time and then decide for each one which is the more relevant thing! Today was a challenge because I was saying to myself I have to use the three hats and do it quick. I was happy with the way I was managing that today*. Migrant and Community interpreter IRL SE
	*Our continuing session [Direct Ranking]… flowed together with this methodology used, because we did not forget our previous commentary [charts] based on this participatory approach*. GR Primary care nurse SE
	*It [Direct Ranking] was just wonderful, it was the high point of the whole research [process in Stage 2]… it flowed, everybody was enjoying it [and] everybody got absolutely involved in the whole business*. ENG Researcher #1 R Int
	*The ranking was interactive. It was an important thing we found as we saw that people during the ranking were already interacting*—*”hey, what are we going to do?”*—*and that was quite a natural process. It was not a process of winning and losing, I found out. That was surprising to me*. NL Researcher #1 R Int
3. Democracy‐in‐action	*20 votes rather than one*—*very interesting! Colour‐coded voting [was] excellent. [A] visual as well as numeric result!* IRL Health service planner PE
	*The best part for me was the voting process, everything was equal*. GR Migrant SE
	*The process of voting [during Direct Ranking] and the result itself… I think this really helped them to [have] trust in the technique*. AUS Researcher #1 R Int
	*This was a qualitative technique which resolved into a quantitative technique, kind of… you can also really give them a number. You can say, okay, out of 10 people, eight liked that [GTI] the most… and I really think our stakeholders, and especially I have to say our GP, really liked this also the most*. AUS Researcher #1 R Int
	*And it was fantastic I think to see how well the voting process actually worked and you know when the final figures were tallied that in fact all of the stakeholders were very, very happy with the outcome. That was striking… a very positive outcome because it needn't have gone that way*. IRL Researcher #1 R Int
	*Yeah, it's also good for people with less language skills, it's a good system for them to be part of the process… it's about working with paper clips and it's visual and I thought especially the ranking process made them [migrant stakeholders] part of the whole group, because in the beginning it was difficult for some migrants to express themselves. And then, they were, you know, standing next to each other and doing it by themselves and it [the process] was really helpful to get them committed, in my opinion*. NL Researcher #1 R Int
4. Power, ownership	*I feel like we have accomplished so much and this methodology shows it!* GR GP SE
	*It gave me a feeling of importance to participate here*. AUS Migrant SE
	*Yes, it [choosing the GTI] matters! We looked at options, positive/negative, and together we came up with a decision. It is important as we go to the next stage [Stage 3 of research] that we “own” the option chosen*. IRL Health Service Planner PE
	*But there was another training [GTI] that was exclusively for GPs that could be very good and strong training [which] was not appropriate for other parties, so the GPs, although they would have liked that one themselves, they chose the other one, which they could be involved in as well*. NL Researcher #2 R Int
	*Both of our [stakeholder] groups were very enthusiastic… we explained it [voting system] and they felt that they had power, it's kind of like how you taught us… it's giving them power, the votes, so we saw that. And what else? They were excited*. GR Researcher #2
Sustained engagement	*And also I think they found it fun, the stakeholders around the table*—*they were also a bit surprised but they considered it fun*. NL Researcher #2 R Int
	*The process worked so well… every stakeholder was happy with the chosen GTI. That was amazing… it's a great testament to the process. [There] was a great sense of achievement, personal achievement, team achievement and stakeholder group achievement, fantastic. And then… an eagerness to move on to the next stage as well*. IRL Researcher #1 R Int
	*We also think the [PLA] system will work … because people like these methods, they will go further on with this*. NL Researcher #1 R Int

Stakeholders described their overall involvement in the PLA process of co‐generation and co‐analysis in ways that speak powerfully of *(1) meaningful engagement in a “safe space”*: active inclusion, collegiality, collaboration. They reported that group dynamics were positive. The working environment was considered safe, allowing stakeholders to readily and safely express diverse views in a relaxed and enjoyable manner. This was also noted and commented on by researchers.

Stakeholders described how there was *(2a) enhanced learning* throughout the process of using Commentary Charts and Direct Ranking. The Commentary Charts facilitated exposure to each other's perspectives about the GTIs and contributed to positive experiences of enhanced learning. Researchers considered that this placed stakeholders in a more informed position from which to prioritize GTIs during Direct Ranking. For example, in Ireland, details about time demands in general practice surgeries were described by the general practice stakeholders. This enhanced other stakeholders’ understanding of the clinical setting and impacted on their assessment and ranking of the GTIs that they were examining (see File S3 for a detailed example from the Irish setting).

It was clear that *(2b) enhanced learning led to important new understandings* and, on occasion, resulted in shifts away from long‐held positions and towards new possibilities. This suggests that the sharing of diverse perspectives during co‐analysis enabled stakeholders to broaden their horizons. Researchers’ comments reflected stakeholders’ sense of the “flow” between the two techniques. Researchers noted the interactive energy of stakeholders’ involvement in a PLA process that generated a “win‐win” outcome.

Furthermore, the visual nature of Direct Ranking allowed stakeholders to engage in a nuanced decision‐making process which was demonstrably democratic. This indicated *(3) democracy‐in‐action*. Stakeholders enjoyed positioning GTIs and Post‐It notes (stickies) and distributing their 20 “paper clip” votes on the chart. Asymmetrical power relations were balanced in part by the fact that 20 votes per stakeholder meant equal voting power and equal opportunity to influence the outcome. Finally, by arranging the GTI images according to the vote count, the democratic outcome was immediately accessible to all and the chart showed the important result stakeholders had achieved.

Researchers noted the equalizing power of PLA, “levelling the playing field,” and that stakeholders with a lower level of language skills were not disenfranchised—they could “see” the result; the presence (density) compared to absence (paucity) of paper clips visually expressed what a numerical count confirmed.

Stakeholders recognized that their choice represented a fundamental input into the progress and trajectory of the research. Exercising power in this way contributed to a genuine sense of “ownership” of the research project: *(4) power, ownership*. Researchers commented on the genuineness/authenticity of stakeholders’ interactions—the willingness of some stakeholders to put others before themselves. This was even the case where the vested interests of typically powerful stakeholders might be expected to win the day, but where choices inclusive of all stakeholders actually won out.

An important outcome was *(5) sustained engagement*. Direct Ranking provided opportunities for stakeholders to experience “positive power,” and researchers noted that they often linked this to the “light” side of PLA: energy, enjoyment, fun and achievement. This “lightness” seemed to offset research fatigue and contributed to sustained meaningful engagement of stakeholders in Stage 2 research.

Negative comments were also identified. Speed evaluation data from The Netherlands comprised a total of 21 stakeholder comments. Of these, there were five negative responses. Speed and participatory evaluation data from Ireland, where evaluations were more extensive, generated 80 stakeholder comments. There were two negative responses. No negative comments were recorded in data from Greece or Austria. However, our content analysis showed that five of 15 stakeholders in Austria chose not to offer evaluation comments of any nature.

Analysis of the negative comments about the PLA techniques shows that the process was considered overly time‐consuming by some Dutch clinical stakeholders. There was also a reduced sense of inclusion for some stakeholders in The Netherlands due to the interstakeholder representation in the PLA sessions.

1. *“Slow, too labour‐intensive.”* Netherlands (NL) Nurse SE
2. *“Useful meeting, the presentation can be done faster.”* NL General Practitioner SE
3. *“Learning‐full, but I felt a little like an outsider not involved in daily practice.”* NL Practice Manager SE
4. *“Nice! Maybe without migrant participants there might be more space for interaction.”* NL SH#76 SE

The other negative comments were RESTORE‐specific (rather than about the PLA processes per se) and came from The Netherlands and Ireland, early in Stage 2. They related to the view that the summaries of the GTI were too brief and needed to be longer to make better judgements, and a degree of uncertainty about what the objective of the process was.

1. *“I am not sure of the potential benefits of assessing GTIs.”* Ireland (IRL) Community Interpreter SE
2. *“Not clear on the outcome of the session/what we want to achieve by assessing GTIs?”* IRL Community Interpreter SE

## 4 DISCUSSION

In this paper, we have explored the perceived utility of two PLA techniques—Commentary Charts and Direct Ranking—for data generation and analysis with a diverse sample comprising migrants, general practice staff, community interpreters, service providers, service planners and academic researchers.

### 4.1 Summary of findings

Our findings show that Commentary Charts and Direct Ranking techniques, with their visual nature and inherent analytical capabilities, were experienced by stakeholders from both community and health‐care settings as powerful tools for collaborative decision making. There was consensus among stakeholders and researchers that there were few *negative experiences*, and numerous multifaceted *positive experiences* of meaningful engagement: PLA created a “safe space” and a trusting environment in which they learned from each other's perspectives, gained enhanced knowledge via the co‐generation of Commentary Charts and used these data to inform their co‐analysis during Direct Ranking. Using these two PLA techniques involved stakeholders in an experience of “democracy‐in‐action” which was empowering and energizing, promoting a sense of ownership and sustained engagement in the research project.

### 4.2 Contribution to existing literature

There is limited knowledge about suitable methods for involving stakeholders in a meaningful (rather than tokenistic) way in health research partnerships.^3^, ^11^ There is evidence that participatory learning and action research approaches and methods seem promising.^5^, ^32^, ^33^, ^34^, ^35^, ^36^ In line with this, our findings show that the application of specific PLA techniques in RESTORE proved fit for purpose. Taken together, the five themes show that academic researchers and stakeholders from community and health sector backgrounds reported that it facilitated collaborative decision making and meaningful engagement, automatically incorporating co‐generation and co‐analysis of data by diverse stakeholders operating in diverse primary care settings.

Furthermore, we provide new evidence about *how* PLA techniques are capable of delivering this—their combined visual, verbal and tangible nature and inherently analytical capabilities involved stakeholders in structured, integrated co‐generation and co‐analysis of research data, which delivered practical democratic results at all five sites. This empirical evidence about the capacity of PLA to “deliver” meaningful engagement in data generation and co‐analysis is a significant addition to a literature that calls for methodological innovation in this sphere. These findings provide important empirical data about *experiences of participation* that are lacking in the literature.^3^ They support Kothari's assertion^9^ that the types of techniques used by researchers can constrain or enable stakeholder involvement (eg findings from Theme 1 that the use of PLA created a safe and meaningful space for engagement and from Theme 5 that it led to sustained engagement for the duration of the fieldwork). They are also in line with Brett et al.,^1^ elucidating that involving stakeholders in data analysis ensures a broader interpretation of data (eg findings from Theme 2 about enhanced learning).

In keeping with Tierney et al.,^5^ we actively explored both positive and negative experiences. In line with Domecq et al.'s review,^11^ stakeholders were predominantly positive about their experiences. In particular, themes 3 and 4 (Democracy‐in‐action; Power, ownership) reveal the ways in which power imbalances were reduced and that experiential learning, rather than reiteration of professional concepts, became the *common ground* upon which democratic decision making took place. This is an important function of PLA: to “level the playing field” where asymmetric power relations between stakeholders/stakeholder groups may exist.^7^

Our results show that this levelling also occurred between stakeholders and researchers: throughout the Commentary Charts and Direct Ranking, it was stakeholders who exercised power and took on the key responsibility of selecting a GTI for implementation, thereby “setting the agenda” for the final stage of RESTORE fieldwork. Researchers, in their capacity as catalysts, facilitated but did not control this process. This shared ownership and agenda‐setting takes us firmly beyond tokenism and towards a “shared power” approach, enhancing the research partnership.

The rare, negative experiences reported by stakeholders in this study warrant attention: the time and pace of research, the need for comprehensive information to facilitate equitable participation, and the possibility that bringing different types of stakeholders together may, on occasion, actually reduce a sense of active inclusion for a minority. While concerns about time demands and tokenistic participation have been reported previously,^11^ the empirical findings about disadvantages of interactions in mixed stakeholder groups are new. Therefore, this analysis of two PLA techniques to support such dialogues is an important contribution to the literature.

### 4.3 Methodological critique and suggestions for future research

We were unable, for site‐specific ethical reasons, to include the use of stakeholder evaluation comments from the English site in our thematic and content analyses. However, we were able to ameliorate this by including comments from researchers’ reflection interviews.

We cannot claim representativeness of findings for the qualitative study data presented here. However, we emphasize that in this comparative case study, spanning five European countries with very different primary care systems, the same PLA techniques were used and successfully involved diverse stakeholders in data generation and co‐analysis.

Regarding the PLA techniques employed, there was variation in our use of PLA Commentary Charts: stakeholders reviewed the Commentary Charts together at the same time in some settings, while some were physically removed from one another and reviewing the Commentary Charts after others had done so. This may have created variation across the sites and this possibility would be worth exploring in future work.

Regarding evaluation methods employed: speed evaluations are efficient and the key messages recorded were similar to those in the additional participatory evaluation conducted at the Irish site. However*,* the brevity of speed evaluations does not encourage rich, lengthy, in‐depth stakeholder responses. The additional participatory evaluation conducted did provide richer data, while researchers’ reflections also added to the quality and depth of evaluation data. Therefore, we suggest using an array of PLA evaluation techniques in future projects to explore all stakeholders’ experiences of involvement in greater depth.

RESTORE ended before our thematic analysis of stakeholders’ evaluation data took place, and we did not, therefore, benefit from their contribution to the development of codes and categories for thematic analysis, nor from their potential insights about the relevance and veracity of evaluation results. While we are confident that data saturation was achieved as the analysis reached a point where the codes and themes were comprehensive, we acknowledge the lack of member checking. In future projects, it would be apposite to invite stakeholders to co‐generate evaluation criteria and to co‐analyse the results of evaluation data, thus closing the circle of “involvement.”

To add to the evidence base, we need further research and evaluation to explore whether and how PLA techniques might work when applied in projects with very different research foci and stakeholder groups to those in RESTORE. The specifics of involving community and health sector partners in analysis of other qualitative methods, such as interviews and focus groups, would also be valuable.

## 5 CONCLUSION

PLA Commentary Charts and Direct Ranking techniques, with their distinctive visual, verbal and tangible nature and inherently co‐analytical capabilities, are rated very positively by stakeholders and researchers. The positive benefits gained from the PLA process in this study (knowledge sharing, knowledge enhancement, levelling the playing field, new knowledge impacting on collaborative decision making) outweighed the negatives. The significant additional investment of resources was impactful and was worth the time and effort. Therefore, we recommend the use of these two PLA techniques as material practices to enable collaborative decision making and meaningful engagement in health research partnerships.

## CONFLICT OF INTEREST

None to declare.
