# Supplementary material for: Material practices for meaningful engagement: An analysis of participatory learning and action research techniques for data generation and analysis in a health research partnership
Source: Health Expect. 2017 Aug 25;21(1):159–70. doi: 10.1111/hex.12598 (PMC5750692; doi:10.1111/hex.12598)
Supplement: Supplementary file 1 [file HEX-21-159-s001.docx]

**Supplementary File 1. Description of Primary Care Settings in RESTORE fieldwork**

|  | **Austria*** | **Ireland** | **Greece** | **Netherlands** | **UK** |
| --- | --- | --- | --- | --- | --- |
| **General practice location** | Located across the largest city in Austria | Located in a central location in the fourth most populous urban area in Ireland. Workload is half and half private vs public patients | Located in a village in a sparsely populated rural area | Located in a deprived area in the fourth largest city in The Netherlands | Located in a central location with high levels of ethnic diversity and deprivation, in the fifth largest metropolitan area in the UK |
| **Migrant patients** | Varied percentage of migrants from central or eastern European countries across the participating general practices | Approximately 17% of patients are migrants from different origins in Europe and Africa | Large percentage of migrant patients (predominately economic migrants) Various origins, mainly from Albania, Pakistan and Syria | Large percentage of migrant patients from different origins, mainly Turkish, Moroccan and Greek | Wide variety of ethnic backgrounds: 15 patient languages other than English are noted on the practice database |
| **Practice staff (clinical)** | 5 GPs, 1 Primary care nurse, Single handed GPs | 3 general practitioners, Practice Nurse, Physiotherapist, Dietician, visiting Consultant Chiropodist & Podiatrist, visiting Consultant Paeditircian & Neonatologist | 5 GPs on site and 5 GPs stationed at satellite practices in the region. 3 permanent nurses that are on continuous rotation, a mid-wife, Pediatrician, Dentist, Microbiologist and lab technician. | 5 general practitioners (and two ad interim GPs). 4 practice nurses; part of a larger health centre including a pharmacy, physiotherapy and social welfare services | 5 general practitioners. Additional clinics for asthma, diabetes, hypertension and heart disease, and well man and well woman clinics. Wider team includes a Health Visitor and two practice District Nurses |
| **Practice staff (administration)** | Primary Care Manger | Practice manager and office manager. | Health care director | 5 practice secretaries | 7 reception staff. |
| **^Strength of the primary care system** | Weak | Weak | Weak | Strong | Strong |
| **^Out-of-pocket payments required** | Partial | Yes | Yes | No | No |
| **^Policy guidance on migrant health** | Yes | Yes | Weak | Yes | Yes |
| **^Funding for migrant sensitive services** | Yes | Cut | Cut | Cut | Yes |
| **^Interpreting services available** | No | Yes | No | No | Yes |
| **^Strength of the primary care system** | Weak | Weak | Weak | Strong | Strong |
| **^Out-of-pocket payments required** | Partial | Yes | Yes | No | No |
| **^Policy guidance on migrant health** | Yes | Yes | Weak | Yes | Yes |

*Information from Austria is based on a number of participating practices rather than a single site

^taken from O’Donnell et al Reducing the health care burden for marginalised migrants: the potential role for primary care in Europe’*Health Policy*, 120(5), 495–508, <http://dx.doi.org/10.1016/j.healthpol.2016.03.012>.
