# Supplementary material for: Material practices for meaningful engagement: An analysis of participatory learning and action research techniques for data generation and analysis in a health research partnership
Source: Health Expect. 2017 Aug 25;21(1):159–70. doi: 10.1111/hex.12598 (PMC5750692; doi:10.1111/hex.12598)
Supplement: Supplementary file 2 [file HEX-21-159-s002.docx]

**Supplementary File 2. Topic Guide for In-depth Reflection Interviews with RESTORE Researchers**

1. Taking each positive PLA experience (‘story’) in turn, please describe what the **highlight** and/or **insight** was about…

- first, for you as a researcher/team?

- second, for stakeholders?

1. Taking each challenging PLA experience (‘story’) in turn, please describe what the **problem** or **issue** was about and how it was ***managed***, ***overcome,*** or remained ***unresolved***. Challenges may have been addressed by teams, an individual researcher and/or stakeholders. All of this is relevant.
2. Reflecting on the **positive** **PLA experiences/‘stories’/**(**highlights/insights)** you’ve mentioned, how does this inform your vision for the use of PLA in future primary healthcare research?

- How you think the use of PLA contributed to the primary care research you were undertaking?
- What advantages did PLA offer?
- What kind of difference do you think PLA made to your research?
- When you think of how stakeholders responded to PLA in these stories, how does that affect your vision for PLA in future primary healthcare research?

1. Reflecting on the **challenging PLA experiences/‘stories’/**(**issues/problems**) you’ve mentioned, how does this inform your vision for the use of PLA in future primary healthcare research?

- Do you think the use of PLA detracted from the primary care research you were undertaking?
- Were there disadvantages associated with the use of PLA?
- When you think of how stakeholders responded to PLA in these stories, how does that affect your vision for PLA in future primary healthcare research?

1. Add your own individual/team question/s here …

_______________________________________________________________________
