# Supplementary material for: Material practices for meaningful engagement: An analysis of participatory learning and action research techniques for data generation and analysis in a health research partnership
Source: Health Expect. 2017 Aug 25;21(1):159–70. doi: 10.1111/hex.12598 (PMC5750692; doi:10.1111/hex.12598)
Supplement: Supplementary file 3 [file HEX-21-159-s003.docx]

**Supplementary File 3. Enhanced Learning in the RESTORE PLA Dialogue about G TIs: A worked example from Ireland**

The Irish stakeholders shared and assessed five GTIs which had been identified in a previous mapping exercise of GTIs in RESTORE (see de Brun et al., 2015). There were two GTIs from the Netherlands and one each from Ireland, England and Scotland:

1. *Did I explain it clearly?’ How to communicate with migrants with lower education and less command of the Dutch language* (Neth)
2. *Ears of Babel. Culturally sensitive primary health care* (Neth)
3. *Working with an Interpreter is Easy: Self-Directed Training Package for Health Professionals* (IRL)
4. *Working with Interpreters in Health Settings – Guidelines for Psychologists* (Eng)
5. *Working with an Interpreter: Toolkit – Improving Communication for people who use mental health or learning disability services in Scotland* (Sco)

Over a course of five PLA sessions, stakeholders had the opportunity to share ideas and comments about this set of GTIs and their key points were recorded in the PLA Commentary Charts.

In their discussions, there were clear examples of knowledge exchange as stakeholders shared information from their respective backgrounds. For example, stakeholders from the general practice setting emphasised potential problems with implementing GTIs given the time constraints they faced in general practice surgeries. They highlighted potential challenges that would arise if they tried to implement the Dutch training initiative, designed to improve communication with patients who had low literacy levels (GTI No. 1). They asked “how are GPs going to ‘test’ the patients to find out the literacy level of the person [in the first place]? Stakeholders who were community interpreters were able to highlight for others in the group that the English GTI, that provided guidance for psychologists to work with interpreters (GTI No. 4), was unusual in that it was explicit about the importance of thinking about the wellbeing of interpreters in interpreted consultations. However, the general practice stakeholders had concerns about how the focus on mental health would translate into the Irish GP setting and what implications it would have for time and money at practice level. Similarly, while the toolkit from Scotland (GTI No. 5) had an important emphasis on explaining patients’ rights to them, GP and community interpreters raised concerns about how the guidance would translate into the Irish healthcare system. These are important examples of knowledge enhancement between stakeholders.

Overall, the discussions were characterised by two strong points of consensus. First, there was significant, shared interest among stakeholders in knowledge and guidance about the use of interpreters in healthcare consultations. They all agreed that this reflected a major gap in service provision in Ireland because family members, friends and relatives are used on a daily basis in primary care consultations instead of formal, trained interpreters. The stakeholders understood that there would be challenges in relation to time and money at practice level but they repeatedly discussed and agreed about the need for, and value of, implementing interpreters.

Second, stakeholders from all backgrounds agreed that the GTIs from the Netherlands, while interesting, would require a significant amount of translation and adaptation work before they would be ready to introduce into an Irish primary care setting. They were wary about taking on that task because of the time it may cost them for embedding and implementing the GTI into practice.

After listening to each other’s perspectives, and reviewing their completed PLA Commentary Chart, the stakeholders progressed to the Direct Ranking exercise. Given the extent of their concerns about translation and adaptation work of the Dutch GTIs, they agreed not to include them in their Direct Ranking exercise at all. When they voted on the remaining three GTIs, the Irish GTI– *Working with an Interpreter is Easy: Self-Directed Training Package for Health Professionals* – was ranked first and far higher than the Scottish training manual or the English guideline.

This result reflects the Irish stakeholders’ strong interest in implementing interpreters’ services and their desire to work with a GTI that did not need to be translated or adapted for the Irish healthcare setting.
